# Supplementary material for: Psychological flexibility, cognitive emotion regulation and mental health outcomes among patients with asthma in Pakistan
Source: PeerJ. 2023 Jul 10;11:e15506. doi: 10.7717/peerj.15506 (PMC10340097; doi:10.7717/peerj.15506)
Supplement: Supplemental Information 1 [file peerj-11-15506-s001.docx]

**Table 4**

**Total, direct and indirect effects of Psychological Flexibility on Quality of Life**

|  | *β* | *SE* | *Boot C.I 95%* | |
| --- | --- | --- | --- | --- |
|  |  |  | *LL* | *UL* |
| **Total Effect** |  |  |  |  |
| Psychological Flexibility 🡪Quality of Life | .017 | .005 | .007 | .027 |
| **Direct Effect** |  |  |  |  |
| Psychological Flexibility 🡪 Quality of Life | -.003 | .006 | -.014 | .008 |
| **Indirect Effects** |  |  |  |  |
| Psychological Flexibility 🡪 Catastrophising 🡪 Quality of Life | .124 | .053 | .022 | .232 |
| Psychological Flexibility 🡪 Anxiety 🡪 Quality of Life | .023 | .032 | -.039 | .087 |
| Psychological Flexibility 🡪 Catastrophising 🡪 Anxiety 🡪 Quality of Life | .120 | .030 | .068 | .184 |
